# Quantum Phase Transition in the Coupled-Top Model: From *Z*_2_ to U(1) Symmetry Breaking

**DOI:** 10.3390/e27050474

**Published:** 2025-04-27

**Authors:** Wen-Jian Mao, Tian Ye, Liwei Duan, Yan-Zhi Wang

**Affiliations:** 1 School of Physics and Electronic Information, Anhui Normal University, Wuhu 241002, China; maowenjian@ahnu.edu.cn (W.-J.M.); yetian@anhu.edu.cn (T.Y.); 2 Anhui Province Key Laboratory for Control and Applications of Optoelectronic Information Materials, Anhui Normal University, Wuhu 241002, China; 3 Department of Physics, Zhejiang Normal University, Jinhua 321004, China

**Keywords:** coupled-top model, anisotropic coupling, quantum phase transition, quantum criticality, Goldstone mode, mean-field approach, Holstein–Primakoff transformation

## Abstract

We investigate the coupled-top model, which describes two large spins interacting along both *x* and *y* directions. By tuning coupling strengths along distinct directions, the system exhibits different symmetries, ranging from a discrete $Z_{2}$ to a continuous U(1) symmetry. The anisotropic coupled-top model displays a discrete $Z_{2}$ symmetry, and the symmetry breaking induced by strong coupling drives a quantum phase transition from a disordered paramagnetic phase to an ordered ferromagnetic or antiferromagnetic phase. In particular, the isotropic coupled-top model possesses a continuous U(1) symmetry, whose breaking gives rise to the Goldstone mode. The phase boundary can be well captured by the mean-field approach, characterized by the distinct behaviors of the order parameter. Higher-order quantum effects beyond the mean-field contribution can be achieved by mapping the large spins to bosonic operators via the Holstein–Primakoff transformation. For the anisotropic coupled-top model with $Z_{2}$ symmetry, the energy gap closes, and both quantum fluctuations and entanglement entropy diverge near the critical point, signaling the onset of second-order quantum phase transitions. Strikingly, when U(1) symmetry is broken, the energy gap vanishes beyond the critical point, yielding a novel critical exponent of 1, rather than 1/2 for $Z_{2}$ symmetry breaking. The rich symmetry structure of the coupled-top model underpins its role as a paradigmatic model for studying quantum phase transitions and exploring associated physical phenomena.

## 1. Introduction

Quantum phase transitions have emerged as one of the central topics in modern physics [1,2,3], offering profound insights into the fundamental nature of quantum many-body systems. These transitions, which occur at zero temperature and are driven by quantum fluctuations rather than classical thermal fluctuations, often lead to dramatic changes in the ground-state properties of materials, such as the emergence of new ordered phases and the appearance of exotic quantum states [2]. Understanding quantum phase transitions not only enriches our theoretical knowledge of quantum physics but also holds great promise for technological applications, such as the critical quantum metrology [4,5,6,7,8,9].

In spin interaction systems, numerous prototype models, such as the transverse-field Ising model (TFIM), XY model, and their generalizations [10,11,12,13,14,15,16,17], have been extensively studied to explore quantum phase transitions in both the ground states and dynamics. Recently, several approaches involving anisotropic coupling, such as the Monte Carlo method, mean-field theory, and feedback-based quantum algorithms, have attracted attention for investigating phase transition phenomena [18,19,20,21]. Specifically, the anisotropic XY model [18], the anisotropic two-dimensional Ising case with triangular or rectangular lattice [19,20], and the anisotropic next-nearest-neighbor Ising model [21] provide insights into the system’s symmetries and critical properties.

When spin-1/2s in the TFIM are replaced with large spins, we obtain the coupled-top model (CTM) [22,23,24,25,26]. As a paradigmatic bipartite system, the CTM is employed to study the interaction between two large spins. Persistent investigations of dynamical and statistical behaviors in the CTM, including ergodic behavior, quantum scars, and level spacing distribution [23,24,25], have been conducted. In particular, various types of quantum phase transitions in the CTM and its generalized cases [25,27,28,29,30], such as the dynamical, ground-state, and excited-state phase transitions, have attracted significant attention. The detection of novel physical phenomena can be effectively carried out using the out-of-time-order correlator [31,32], which is experimentally implementable [33,34]. Recently, the triangular coupled-top model has emerged as a new platform for studying unconventional frustrated magnetic behaviors, exhibiting phenomena that merit further investigation [35].

Unlike the TFIM or XY model, which is typically studied on a one-dimensional spin chain or higher-dimensional lattices, the coupled-top model is a bipartite system. This model is more analogous to the finite-component light-matter interaction systems [36,37,38,39,40], undergoing the second-order quantum phase transitions from the disorder to ordered phase [41,42,43,44]. Paradigmatic models, such as Rabi, Dicke, and spin-boson model, allow control over symmetries either $Z_{2}$ or U(1) by tuning anisotropic coupling strengths along two orthogonal directions [45,46,47,48,49]. Notably, in systems with either symmetry preservation or breaking, anisotropic matter–light interactions induce novel phase diagrams and quantum triple points [47,48,49], which have received widespread attention. Significantly, these models reduce to the Jaynes–Cummings model [50], Tavis–Cummings model [51], and spin–boson model [52] with rotating wave approximation, accompanied by the symmetry changing from $Z_{2}$ to U(1). The latter exhibits distinct critical behaviors related to the Goldstone mode [53,54]. Similarly, the anisotropy in the coupled-top model can be introduced by adjusting coupling constants along *x*- and *y*-directions separately. This scheme provides an important platform for both experimental and theoretical control of symmetries, as well as for investigating associated physical properties.

In this paper, we investigate quantum phase transitions of the coupled-top model resulting from $Z_{2}$ and U(1) symmetry breaking and the associated quantum criticality. The paper is organized as follows. In Section 2, we define the coupled-top model and explain the $Z_{2}$ and U(1) symmetries. In Section 3, we derive the mean-field solutions and corresponding phase diagram. In Section 4, we extend the analysis beyond the mean-field approach, calculating the excitation energy, quantum fluctuations, entanglement entropy, and critical phenomena. In Section 5, we examine the energy gap related to the Goldstone mode for U(1) symmetry. Finally, Section 6 provides the conclusions of this study.

## 2. The Model and Symmetries

The coupled-top model can be regarded as a generalization of the XY model in a transverse field, with spin-1/2 replaced by large spins. Its Hamiltonian can be written as

(1) $$\begin{array}{c} {\hat{H}}_{\mathrm{ACTM}}=-\epsilon \left({\hat{J}}_{1}^{z}+{\hat{J}}_{2}^{z}\right)+\frac{1}{J}\left(\chi_{x}{\hat{J}}_{1}^{x}{\hat{J}}_{2}^{x}+\chi_{y}{\hat{J}}_{1}^{y}{\hat{J}}_{2}^{y}\right), \end{array}$$

where ${\hat{J}}_{i}^{d}$ denotes the *d*-component (d=x,y,z) collective spin operators of magnitude *J* for the *i*-th large spin. ϵ characterizes the strength of the transverse field. $\chi_{x}$ and $\chi_{y}$ represent coupling constants between spins in the *x*- and *y*-directions, respectively. For clarity, we express the dimensionless Hamiltonian as

(2) $$\begin{array}{c} \hat{H}=\frac{{\hat{H}}_{\mathrm{ACTM}}}{\epsilon }=-\left({\hat{J}}_{1}^{z}+{\hat{J}}_{2}^{z}\right)+\frac{1}{J}\left(\lambda_{x}{\hat{J}}_{1}^{x}{\hat{J}}_{2}^{x}+\lambda_{y}{\hat{J}}_{1}^{y}{\hat{J}}_{2}^{y}\right). \end{array}$$

with coupling constants $\lambda_{x}=\chi_{x}/\epsilon$ and $\lambda_{y}=\chi_{y}/\epsilon$.

Previous studies on the coupled-top model generally consider the interaction in *x*-direction ($\lambda_{y}=0$) [22,23,24,25,30], which is highly anisotropic and only has $Z_{2}$ symmetry. By introducing both $\lambda_{x}$ and $\lambda_{y}$, Hamiltonian (2) becomes much more flexible. Significantly, the generalized coupled-top model bridges the gap between discrete $Z_{2}$ symmetry and continuous U(1) symmetry. We focus on the spontaneous symmetry breaking and critical behaviors associated with quantum phase transitions in the thermodynamic limit (J→+∞).

For $\lambda_{x}=\lambda_{y}$, the coupled-top model is isotropic in the x−y plane. We can easily prove that the Hamiltonian is invariant under an arbitrary rotation along *z* direction, namely, ${\hat{R}}^{†}\left(\alpha \right)\hat{H}\hat{R}\left(\alpha \right)=\hat{H}$, with

(3) $$\begin{array}{c} \hat{R}\left(\alpha \right)=\exp\left[i\alpha \sum_{i=1,2}\left({\hat{J}}_{i}^{z}+J\right)\right],\;\alpha \in \left[0,2\pi \right). \end{array}$$

It indicates that the isotropic coupled-top model possesses the U(1) symmetry.

For $\lambda_{x}\neq \lambda_{y}$, the coupled-top model becomes anisotropic, which exists a $Z_{2}$ symmetry. It manifests the invariance of Hamiltonian under the spin flipping along *x*- and *y*-axis, namely, ${\hat{J}}_{i}^{x}\to -{\hat{J}}_{i}^{x}$, ${\hat{J}}_{i}^{y}\to -{\hat{J}}_{i}^{y}$, and ${\hat{J}}_{i}^{z}\to {\hat{J}}_{i}^{z}$, which corresponds to α=π in Equation (3). Hamiltonian (2) satisfies the commutation relation $[\hat{H},\hat{\Pi }]=0$, with the parity operator

(4) $$\begin{array}{c} \hat{\Pi }=\hat{R}\left(\alpha =\pi \right)=\exp\left[i\pi \sum_{i=1,2}\left({\hat{J}}_{i}^{z}+J\right)\right]. \end{array}$$

For weak coupling without symmetry breaking, the transverse field plays the dominated role, and the coupled-top model tends to be in the paramagnetic phase (PP), with magnetization aligned along the transverse field (*z*-direction). The preserved symmetry leads to $\langle {\hat{J}}_{i}^{x}\rangle =0$ and $\langle {\hat{J}}_{i}^{y}\rangle =0$. In contrast, strong coupling can break the $Z_{2}$ or U(1) symmetry, which leads to the emergence of ferromagnetic phase (FP) or antiferromagnetic phase (AFP). The symmetry breaking is accompanied by the spontaneous magnetization in *x*- or *y*-directions, namely, $\langle {\hat{J}}_{i}^{x}\rangle \neq 0$ or $\langle {\hat{J}}_{i}^{y}\rangle \neq 0$. Therefore, $\langle {\hat{J}}_{i}^{x}\rangle$ and $\langle {\hat{J}}_{i}^{y}\rangle$ serve as the order parameter, which can be employed to distinguish different phases.

## 3. Phase Diagram

To facilitate the analytical study, we first investigate the phase diagram of the coupled-top model in the thermodynamic limit (J→+∞). The mean-field approach, where correlations between large spins are neglected, provides an efficient method to distinguish different phases, especially in the thermodynamic limit (J→+∞) [55,56]. Within the mean-field framework [57,58], we can construct a trial ground state by a tensor product of Bloch coherent states [59],

(5) $$\begin{array}{c} |\psi_{MF}\rangle =|\theta_{1},\phi_{1}\rangle ⊗|\theta_{2},\phi_{2}\rangle =\overset{2}{\underset{i=1}{⨂}}\exp\left[\frac{\theta_{i}}{2}\left(e^{i\phi_{i}}J_{i}^{-}-e^{-i\phi_{i}}J_{i}^{+}\right)\right]|J,J\rangle . \end{array}$$

The expectation values of the large spin operators are represented by the corresponding points on the Bloch sphere as

(6) $$\begin{array}{c} \left(J_{i}^{x},J_{i}^{y},J_{i}^{z}\right)=\frac{\left(\langle {\hat{J}}_{i}^{x}\rangle ,\langle {\hat{J}}_{i}^{y}\rangle ,\langle {\hat{J}}_{i}^{z}\rangle \right)}{J}=\left(\sin\theta_{i}\cos\phi_{i},\sin\theta_{i}\sin\phi_{i},\cos\theta_{i}\right), \end{array}$$

with $\left(0\leq \theta_{i}\leq \pi ,0\leq \phi_{i}<2\pi \right)$, which can be employed to derive the averaged energy expectation value of the system,

(7) $$\begin{array}{cc} E_{\mathrm{MF}} & =\frac{1}{J}\langle \psi_{MF}|\hat{H}|\psi_{MF}\rangle \\ \; & =-\left(J_{1}^{z}+J_{2}^{z}\right)+\lambda_{x}{\hat{J}}_{1}^{x}{\hat{J}}_{2}^{x}+\lambda_{y}{\hat{J}}_{1}^{y}{\hat{J}}_{2}^{y}\\ \; & =-\left(\cos\theta_{1}+\cos\theta_{2}\right)+\lambda_{x}\sin\theta_{1}\cos\phi_{1}\sin\theta_{2}\cos\phi_{2}\\ \; & \;+\lambda_{y}\sin\theta_{1}\sin\phi_{1}\sin\theta_{2}\sin\phi_{2}. \end{array}$$

The variational parameters $\theta_{i}$ and $\phi_{i}$ can be determined by minimizing the energy expectation value given in Equation (7). Applying the variational principle, we can achieve the following equations:

(8a) $$\begin{array}{cc} \; & \frac{\partial E_{\mathrm{MF}}}{\partial \theta_{1}}=\sin\theta_{1}+\lambda_{x}\cos\theta_{1}\cos\phi_{1}\sin\theta_{2}\cos\phi_{2}+\lambda_{y}\cos\theta_{1}\sin\phi_{1}\sin\theta_{2}\sin\phi_{2}=0, \end{array}$$

(8b) $$\begin{array}{cc} \; & \frac{\partial E_{\mathrm{MF}}}{\partial \theta_{2}}=\sin\theta_{2}+\lambda_{x}\sin\theta_{1}\cos\phi_{1}\cos\theta_{2}\cos\phi_{2}+\lambda_{y}\sin\theta_{1}\sin\phi_{1}\cos\theta_{2}\sin\phi_{2}=0, \end{array}$$

(8c) $$\begin{array}{cc} \; & \frac{\partial E_{\mathrm{MF}}}{\partial \phi_{1}}=-\lambda_{x}\sin\theta_{1}\sin\phi_{1}\sin\theta_{2}\cos\phi_{2}+\lambda_{y}\sin\theta_{1}\cos\phi_{1}\sin\theta_{2}\sin\phi_{2}=0, \end{array}$$

(8d) $$\begin{array}{cc} \; & \frac{\partial E_{\mathrm{MF}}}{\partial \phi_{2}}=-\lambda_{x}\sin\theta_{1}\cos\phi_{1}\sin\theta_{2}\sin\phi_{2}+\lambda_{y}\sin\theta_{1}\sin\phi_{1}\sin\theta_{2}\cos\phi_{2}=0. \end{array}$$

**Figure 1:**
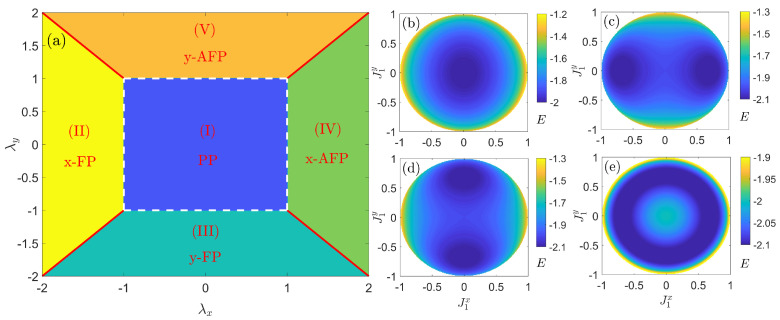
(**a**) Phase diagram in the $\left(\lambda_{x},\lambda_{y}\right)$ plane. The energy surface $E_{\mathrm{MF}}$ by the mean-field approach in four regions: (**b**) the PP region for $\lambda_{x}=0.4$ and $\lambda_{y}=0.7$ with the energy minimum in the origin; (**c**) the *x*-AFP region with two minima in the *x*-axis, where the symmetry is broken along the *x*-direction for $\lambda_{x}=1.4$ and $\lambda_{y}=0.7$; (**d**) the *y*-AFP region with two minima in the *y*-axis, where the symmetry is broken along the *y*-direction for $\lambda_{x}=0.7$ and $\lambda_{y}=1.4$; (**e**) the U(1) symmetry-breaking region for $\lambda_{x}=\lambda_{y}=1.4$ with a circular valley of infinite degenerate minima.

Solving Equations (8a)–(8d) self-consistently, we can achieve $\theta_{i}$ and $\phi_{i}$, as well as the ground-state energy $E_{\mathrm{MF}}^{\min}$ and order parameters $J_{i}^{x,y}$. According to distinct behaviors of the order parameters, the phase diagram is shown in Figure 1a, and the corresponding phase transition points are identified as

(9) $$\begin{array}{c} \lambda_{x}^{c\mp }=\mp 1,\;\lambda_{y}^{c\mp }=\mp 1. \end{array}$$

The quantum phase transitions along the *x*-direction occur at $\lambda_{x}^{c\mp }$, corresponding to transitions from PP to FP and AFP, respectively. Similar behaviors are observed along the *y*-direction with respect to $\lambda_{y}^{c\mp }$. The white dashed lines separate the disordered phase (PP) from ordered phases (FP and AFP), while the red solid lines ($|\lambda_{x}\left|=\right|\lambda_{y}|>1$) distinguish between FP and AFP. Notably, each point along the red solid lines breaks the continuous U(1) symmetry and exhibits an infinite degeneracy. Overall, we classify the ground states into five phases. To visualize behaviors of the ground states in different phase, we display the energy surface $E_{\mathrm{MF}}$ [Equation (7)] as a function of $J_{1}^{x}$ and $J_{1}^{y}$ in Figure 1b–e, whose minimum corresponds to the ground state. A detailed analysis of their physical properties is presented below:

(i) The symmetry-preserving region, where the disordered paramagnetic phase (PP) dominates, occupies the center of the phase diagram, with $\lambda_{d}^{c-}<\lambda_{d}<\lambda_{d}^{c+}$(d=x,y). The PP can be characterized by the analytical solutions

(10) $$\begin{array}{c} \theta_{1}=\theta_{2}=0,\;E_{\mathrm{MF}}^{\min}=-2. \end{array}$$

As evidenced in Figure 1b, the numerical energy minimum resides at $\left(J_{i}^{x},J_{i}^{y}\right)=\left(0,0\right)$ for $\lambda_{x}=0.4$ and $\lambda_{y}=0.7$, confirming the theoretical predictions.

(ii) The $Z_{2}$ symmetry-breaking region along the *x*-direction arises when the coupling strength $\lambda_{x}$ exceeds the critical value while maintaining $|\lambda_{x}|>|\lambda_{y}|$. This region can be described by

(11) $$\begin{array}{c} \theta_{1}=\theta_{2}=\arccos\left(\frac{1}{|\lambda_{x}|}\right),\;E_{\mathrm{MF}}^{\min}=-\frac{\lambda_{x}^{2}+1}{|\lambda_{x}|}. \end{array}$$

Specifically, for $\lambda_{x}<\lambda_{x}^{c-}$, the ordered ferromagnetic phase (*x*-FP) dominates with

(12) $$\begin{array}{c} \phi_{1}=\phi_{2}=0\;\;or\;\;\pi ,\;J_{1}^{x}=J_{2}^{x}=\pm \sqrt{1-\frac{1}{\lambda_{x}^{2}}},\;J_{i}^{y}=0. \end{array}$$

For $\lambda_{x}>\lambda_{x}^{c+}$, the system stabilizes in the ordered antiferromagnetic phase (*x*-AFP) where

(13) $$\begin{array}{c} \phi_{i}=0,\;\phi_{i+1}=\pi ,\;J_{1}^{x}=-J_{2}^{x}=\pm \sqrt{1-\frac{1}{\lambda_{x}^{2}}},\;J_{i}^{y}=0. \end{array}$$

Notably, the two possible values of $\phi_{i}$ induce a double degeneracy in both the FP and AFP. This is consistent with numerical results shown in Figure 1c, where the energy displays two minima, one with a positive and the other with a negative value, along the $J_{1}^{x}$ axis for $\lambda_{x}=1.4$ and $\lambda_{y}=0.7$.

(iii) The $Z_{2}$ symmetry-breaking region along the *y*-direction occurs when $\lambda_{y}$ varies and crosses the critical value under the condition $|\lambda_{y}|>|\lambda_{x}|$. This regime is governed by the following:

(14) $$\begin{array}{c} \theta_{1}=\theta_{2}=\arccos\left(\frac{1}{|\lambda_{y}|}\right),\;E_{\mathrm{MF}}^{\min}=-\frac{\lambda_{y}^{2}+1}{|\lambda_{y}|}. \end{array}$$

Clearly, for $\lambda_{y}<\lambda_{y}^{c-}$, the ordered ferromagnetic phase (*y*-FP) is dominant with

(15) $$\begin{array}{c} \phi_{1}=\phi_{2}=\frac{\pi }{2}\;\;or\;\;\frac{3\pi }{2},\;J_{i}^{x}=0,\;J_{1}^{y}=J_{2}^{y}=\pm \sqrt{1-\frac{1}{\lambda_{y}^{2}}}. \end{array}$$

For $\lambda_{y}>\lambda_{y}^{c+}$, the system enters the ordered antiferromagnetic phase (*y*-AFP) where

(16) $$\begin{array}{c} \phi_{i}=\frac{\pi }{2},\;\phi_{i+1}=\frac{3\pi }{2},\;J_{i}^{x}=0,\;J_{1}^{y}=-J_{2}^{y}=\pm \sqrt{1-\frac{1}{\lambda_{y}^{2}}}. \end{array}$$

Analogous to the *x*-direction case, a two-fold degeneracy with dual minima takes place along the *y*-axis, which is visualized in Figure 1d for $\lambda_{x}=0.7$ and $\lambda_{y}=1.4$.

(iv) The U(1) symmetry-breaking region emerges beyond the critical points (indicated by red solid lines in Figure 1 for $|\lambda_{x}|=|\lambda_{y}|=|\lambda |>1$). This region is analytically defined by:

(17) $$\begin{array}{c} \theta_{1}=\theta_{2}=\arccos\left(\frac{1}{\left|\lambda \right|}\right),\;E_{\mathrm{MF}}^{\min}=-\frac{\lambda^{2}+1}{\left|\lambda \right|}. \end{array}$$

Crucially, the infinite degeneracy arises from the continuous freedom of $\phi_{i}\in \left[0,2\pi \right)$, with the following conditions: $\phi_{1}-\phi_{2}=\pi$ for $\lambda_{x}=\lambda_{y}=\lambda >0$, $\phi_{1}+\phi_{2}=2\pi$ for $\lambda_{x}=-\lambda_{y}=\lambda <0$, $\phi_{1}-\phi_{2}=0$ for $\lambda_{x}=\lambda_{y}=\lambda <0$, and $\phi_{1}+\phi_{2}=\pi$ for $\lambda_{x}=-\lambda_{y}=\lambda >0$, respectively. As shown in Figure 1e for $\lambda_{x}=\lambda_{y}=1.4$, the ground states form a circular manifold of energy minima resembling a Mexican hat profile. All spin polarizations align uniformly in the θ-direction while permitting continuous $\phi_{i}$ variations, a hallmark of continuous symmetry breaking.

Above all, by adjusting coupling strength $\lambda_{x}$ and $\lambda_{y}$, we achieve precise control over the conservation and breaking of $Z_{2}$ and U(1) symmetries. This capability establishes a promising platform for investigating quantum phase transitions and their associated emergent phenomena.

## 4. Quantum Criticality

Although the mean-field approach successfully produces the phase diagram, certain critical behaviors in the vicinity of the phase transition points remain hidden. This is because these behaviors are associated with higher-order contributions that lie outside the scope of the mean-field approximation. To systematically address these effects, we first apply a unitary transformation [35,57,60,61,62], $\hat{\tilde{H}}={\hat{U}}^{†}\hat{H}\hat{U}$, with

(18) $$\begin{array}{ccc} \hat{U} & = & {\hat{U}}_{1}⊗{\hat{U}}_{2}, \end{array}$$

(19) $$\begin{array}{ccc} {\hat{U}}_{i} & = & \exp\left(-i\phi_{i}{\hat{J}}_{i}^{z}\right)\exp\left(-i\theta_{i}{\hat{J}}_{i}^{y}\right),\;i=1,2, \end{array}$$

where $\phi_{i}$ and $\theta_{i}$ are the mean-field solutions from Section 3. This unitary transformation rotates spins sequentially around the *z*-axis with angles $\phi_{i}$ followed by rotations about *y*-axis by angles $\theta_{i}$. The spin operators in the transformed Hamiltonian $\hat{\tilde{H}}$ can be expressed as

(20) $${\hat{U}}_{i}^{†}\left(\begin{array}{c} {\hat{J}}_{i}^{x}\\ {\hat{J}}_{i}^{y}\\ {\hat{J}}_{i}^{z} \end{array}\right){\hat{U}}_{i}=\left(\begin{array}{ccc} \cos\theta_{i}\cos\varphi_{i} & -\sin\varphi_{i} & \sin\theta_{i}\cos\varphi_{i}\\ \cos\theta_{i}\sin\varphi_{i} & \cos\varphi_{i} & \sin\theta_{i}\sin\varphi_{i}\\ -\sin\theta_{i} & 0 & \cos\theta_{i} \end{array}\right)\left(\begin{array}{c} {\hat{J}}_{i}^{x}\\ {\hat{J}}_{i}^{y}\\ {\hat{J}}_{i}^{z} \end{array}\right).$$

Subsequently, we introduce the mapping from the spin operators to the bosonic creation and annihilation operators via Holstein–Primakoff transformation [63]. In the thermodynamic limit (J→+∞) where $\;J\gg \langle {\hat{a}}_{i}^{†}{\hat{a}}_{i}\rangle$, the transformation is expressed as

(21a) $$\begin{array}{cc} {\hat{J}}_{i}^{z} & =J-{\hat{a}}_{i}^{†}{\hat{a}}_{i}, \end{array}$$

(21b) $$\begin{array}{cc} {\hat{J}}_{i}^{+} & =\sqrt{2J-{\hat{a}}_{i}^{†}{\hat{a}}_{i}}{\hat{a}}_{i}\approx \sqrt{2J}\;{\hat{a}}_{i}, \end{array}$$

(21c) $$\begin{array}{cc} {\hat{J}}_{i}^{-} & ={\hat{a}}_{i}^{†}\sqrt{2J-{\hat{a}}_{i}^{†}{\hat{a}}_{i}}\approx \sqrt{2J}\;{\hat{a}}_{i}^{†}, \end{array}$$

where the bosonic operators satisfy $\left[{\hat{a}}_{i},{\hat{a}}_{j}^{†}\right]=\delta_{ij}$.

By combining Equations (20) and (21), we can expand the Hamiltonian $\hat{\tilde{H}}$ and neglect the terms of order $J^{-s}$ with s>0 due to the limit (J→+∞), yielding:

(22) $$\hat{\tilde{H}}=J^{1}E_{\mathrm{MF}}+J^{\frac{1}{2}}{\hat{\tilde{H}}}_{1}+J^{0}{\hat{\tilde{H}}}_{2}.$$

The first term $E_{\mathrm{MF}}$ represents the ground-state energy obtained from the mean-field approach. The second term can be eliminated, namely, ${\hat{\tilde{H}}}_{1}=0$ in Equation (A1), as $\theta_{i}$ and $\phi_{i}$ satisfy Equations (8a)–(8d). The third term ${\hat{\tilde{H}}}_{2}$ has a quadratic form, as expressed in Equation (A2) in Appendix A. It can be further simplified by substituting $\theta_{i}$ and $\phi_{i}$ in different phases given in Section 3. For example, by substituting the values of $\theta_{i}$ and $\phi_{i}$ for the *x*- and *y*-direction symmetry breaking, we can simplify the general form of ${\hat{\tilde{H}}}_{2}$ in Equation (A2) as

(23) $$\begin{array}{cc} {\hat{\tilde{H}}}_{2}= & \;\left(\cos\theta_{1}+\left|\lambda_{d}\right|\sin\theta_{1}\sin\theta_{2}\right){\hat{a}}_{1}^{†}{\hat{a}}_{1}\\ \; & +\left(\cos\theta_{2}+\left|\lambda_{d}\right|\sin\theta_{1}\sin\theta_{2}\right){\hat{a}}_{2}^{†}{\hat{a}}_{2}\\ \; & -\frac{1}{2}\left|\lambda_{d}\right|\cos\theta_{1}\cos\theta_{2}\left({\hat{a}}_{1}^{†}+{\hat{a}}_{1}\right)\left({\hat{a}}_{2}^{†}+{\hat{a}}_{2}\right)\\ \; & +\frac{1}{2}f\lambda_{d^{\prime }}\left({\hat{a}}_{1}^{†}-{\hat{a}}_{1}\right)\left({\hat{a}}_{2}^{†}-{\hat{a}}_{2}\right), \end{array}$$

where $|\lambda_{d}\left|>\right|\lambda_{d^{\prime }}|$. It indicates that the index d=x,y designates the principal symmetry-breaking axis, while $d^{\prime }=y,x$ marks the orthogonal direction with preserved symmetry. The discrete parameter f=∓1 serves as a region indicator, corresponding to the regimes $\lambda_{d}<0$ and $\lambda_{d}>0$, respectively. Furthermore, Equation (23) is also suitable to the symmetry-preserving PP region, which corresponds to $\theta_{1}=\theta_{2}=0$.

To systematically diagonalize the quadratic Hamiltonian ${\hat{\tilde{H}}}_{2}$, we employ the $\hat{x}-\hat{p}$ representation by constituting the canonical vector $\hat{r}={\left({\hat{x}}_{1},{\hat{x}}_{2},{\hat{p}}_{1},{\hat{p}}_{2}\right)}^{T}$ with ${\hat{x}}_{i}=\left({\hat{a}}_{i}^{†}+{\hat{a}}_{i}\right)/\sqrt{2}$ and ${\hat{p}}_{i}=i\left({\hat{a}}_{i}^{†}-{\hat{a}}_{i}\right)/\sqrt{2}$. $\hat{r}$ satisfies the canonical commutation relations, $\left[\hat{r},{\hat{r}}^{T}\right]=i\hat{\Gamma }$, where $\Gamma =\left(\begin{array}{cc} O_{2} & I_{2}\\ -I_{2} & O_{2} \end{array}\right),$ with $I_{2}$ and $O_{2}$ the 2×2 identity and null matrices, respectively. Then, the quadratic Hamiltonian ${\hat{\tilde{H}}}_{2}$ can be rewritten as

(24) $$\begin{array}{cc} {\hat{\tilde{H}}}_{2} & =\sum_{i=1,2}\frac{\omega_{i}}{2}\left({\hat{x}}_{i}^{2}+{\hat{p}}_{i}^{2}-1\right)+\tau_{i,i+1}{\hat{x}}_{i}{\hat{x}}_{i+1}+g_{i,i+1}{\hat{p}}_{i}{\hat{p}}_{i+1}\\ \; & =\frac{1}{2}{\hat{r}}^{T}H\hat{r}-\sum_{i=1,2}\frac{\omega_{i}}{2}, \end{array}$$

with $H=H_{x}⊕H_{p}$ and

(25) $$H_{x}=\left(\begin{array}{cc} \omega_{1} & \tau_{1,2}\\ \tau_{2,1} & \omega_{2} \end{array}\right),\;H_{p}=\left(\begin{array}{cc} \omega_{1} & g_{1,2}\\ g_{2,1} & \omega_{2} \end{array}\right).$$

Based on the Williamson theorem [64], for the positively defined real matrix *H*, we can find a symplectic transformation *S* ($S^{T}\Gamma S=\Gamma$) such that

(26) $$S^{T}HS=\Lambda ,\;\mathrm{with}\;\Lambda =\mathrm{diag}\left(\Delta_{1},\Delta_{2},\Delta_{1},\Delta_{2}\right).$$

In virtue of the symplectic transformation *S*, we can construct a new vector of canonical operators ${\hat{r}}^{\prime }=S^{-1}\hat{r}$, with which the quadratic Hamiltonian ${\hat{\tilde{H}}}_{2}$ can be decoupled into two independent harmonic oscillators, as follows:

(27) $$\begin{array}{cc} {\hat{\tilde{H}}}_{2} & =\sum_{i=1,2}\frac{\Delta_{i}}{2}\left({\hat{x}}_{i}^{\prime 2}+{\hat{p}}_{i}^{\prime 2}\right)-\sum_{i=1,2}\frac{\omega_{i}}{2}\\ \; & =\frac{1}{2}{\hat{r}}^{\prime T}\Lambda {\hat{r}}^{\prime }-\sum_{i=1,2}\frac{\omega_{i}}{2}. \end{array}$$

In the symmetry-breaking regions where $\theta_{1}=\theta_{2}=\arccos\left(\frac{1}{|\lambda_{d}|}\right)$, above parameters are given by $\omega =\omega_{1}=\omega_{2}=\left|\lambda_{d}\right|$, $\tau =\tau_{1,2}=\tau_{2,1}=-\frac{1}{|\lambda_{d}|}$ and $g=g_{1,2}=g_{2,1}=-f\lambda_{d^{\prime }}$. In contrast, in the symmetry-preserving PP region where $\theta_{1}=\theta_{2}=0$, the parameters simplify to $\omega =\omega_{1}=\omega_{2}=1$, $\tau =\tau_{1,2}=\tau_{2,1}=-\left|\lambda_{d}\right|$ and $g=g_{1,2}=g_{2,1}=-f\lambda_{d^{\prime }}$.

Moreover, using the symplectic transformation *S*, we can construct 4×4 covariance matrix σ [35,60,64,65], given by $\sigma =\frac{1}{2}\langle \left\{\left(\hat{r}-\langle \hat{r}\rangle \right),{\left(\hat{r}-\langle \hat{r}\rangle \right)}^{T}\right\}\rangle =\frac{1}{2}SS^{T}$. The quantum fluctuations ${\left(\Delta x_{i}\right)}^{2}$ in position space and ${\left(\Delta p_{i}\right)}^{2}$ in momentum space are defined by the diagonal elements of the covariance matrix, namely ${\left(\Delta x_{i}\right)}^{2}=\langle {\hat{x}}_{i}^{2}\rangle -{\langle {\hat{x}}_{i}\rangle }^{2}=\sigma_{i,i}$ and ${\left(\Delta p_{i}\right)}^{2}=\langle {\hat{p}}_{i}^{2}\rangle -{\langle {\hat{p}}_{i}\rangle }^{2}=\sigma_{2+i,2+i}$ for i=1,2.

Close to distinct quantum phase transition points $\lambda_{d}^{c-}=-1$ and $\lambda_{d}^{c+}=+1$, we can analytically compute the the excitation energy $\Delta_{i}$, quantum fluctuations ${\left(\Delta x_{i}\right)}^{2}$ and ${\left(\Delta p_{i}\right)}^{2}$. The explicit expressions for these quantities in both the symmetry-breaking and the symmetry-preserving paramagnetic phases are derived in Appendix B; see in particular Equations (B3) and (B4).

The entanglement entropy [35,60,65,66,67], serving as a measure of the entanglement between distinct subsystems, is intimately connected to Heisenberg’s uncertainty relation in the context of the quadratic Hamiltonian for interacting bosonic systems. In terms of $\Delta x_{i}$ and $\Delta p_{i}$, the entanglement entropy can be calculated by

(28) $$S_{i}=\left(\Delta x_{i}\Delta p_{i}+\frac{1}{2}\right)\ln\left(\Delta x_{i}\Delta p_{i}+\frac{1}{2}\right)-\left(\Delta x_{i}\Delta p_{i}-\frac{1}{2}\right)\ln\left(\Delta x_{i}\Delta p_{i}-\frac{1}{2}\right).$$

**Figure 2:**
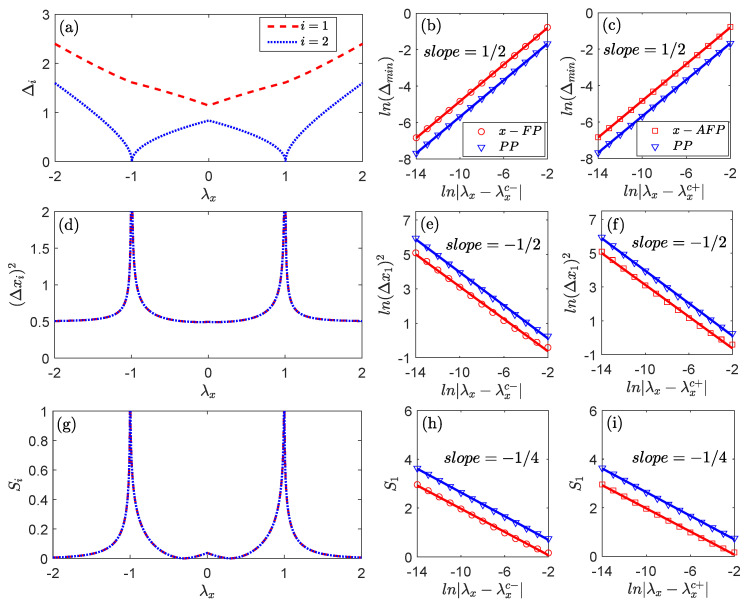
Critical behavior near the phase transition point due to $Z_{2}$ symmetry breaking: (**a**) The excitation energy $\Delta_{i}$; (**d**) the quantum fluctuation ${\left(\Delta x_{i}\right)}^{2}$; (**g**) the entanglement entropy $S_{i}$ as a function of the coupling strength $\lambda_{x}$ for $\lambda_{y}=0.3$. The red dashed line and blue dotted line correspond to the spin index i=1,2, respectively. The critical points are situated at $\lambda_{x}^{c-}=-1$ and $\lambda_{x}^{c+}=+1$, which distinguish the *x*-FP from PP and the PP from *x*-FP, respectively. (**b**,**c**) represent the lowest excitation energy $\Delta_{min}$, and (**e**,**f**) represent the quantum fluctuation ${\left(\Delta x_{1}\right)}^{2}$ as a function of $|\lambda_{x}-\lambda_{x}^{c\mp }|$ near the critical points. (**h**,**i**) show the entanglement entropy $S_{1}$. The red circles and blue triangles correspond to the analytically exact results for the *x*-FP (*x*-AFP) and the PP, respectively, while the solid lines refer to the numerically fitted results. For visibility, the red and blue curves have been shifted to distinguish them.

As shown in Figure 2, we plot the panels for the excitation energy $\Delta_{i}$, the quantum fluctuation ${\left(\Delta x_{i}\right)}^{2}$, and the entanglement entropy $S_{i}$ as functions of the coupling strength $\lambda_{x}$ with $\lambda_{y}=0.5$ held constant. In panel (a), the lowest excitation energy $\Delta_{min}=\min\left(\Delta_{1},\Delta_{2}\right)$, which corresponds to the energy gap of the system, vanishes at the critical points $\lambda_{x}^{c\mp }=\mp 1$. This behavior indicates the second-order quantum phase transitions from the FP to the PP, and from the PP to the AFP, along the *x*-direction. Similarly, the quantum fluctuation ${\left(\Delta x_{i}\right)}^{2}$ in panel (d) and the entanglement entropy $S_{i}$ in panel (g) both exhibit divergences close to the critical points, but tend to stabilize at constant values away from these points. Clearly, these features provide strong evidence for the existence of quantum phase transitions.

All the quantum phase transitions shown in Figure 2 are associated with the $Z_{2}$ symmetry breaking along the *x*-direction. To better understand the universal quantum criticality, it is essential to investigate the critical behaviors of these phase transitions. In panels (b,c), the ln-ln plots of the lowest excitation energy consistently follow the exponential law $\Delta_{min}\propto {\left|\lambda_{x}-\lambda_{x}^{c\mp }\right|}^{1/2}$ near critical points in four regions: $\lambda_{x}\to \lambda_{x}^{c-}$ (*x*-FP), $\lambda_{x}^{c-}\to \lambda_{x}$ (PP), $\lambda_{x}\to \lambda_{x}^{c+}$ (PP), and $\lambda_{x}^{c-}\to \lambda_{x}$ (*x*-FP). The divergences of the quantum fluctuation in panels (e,f) exhibit exponential scaling relations in each region, where ${\left(\Delta x_{i}\right)}^{2}\propto {\left|\lambda_{x}-\lambda_{x}^{c\pm }\right|}^{-1/2}$. Subsequently, we obtain that $S_{i}\propto -\frac{1}{4}\ln\left|\lambda_{x}-\lambda_{x}^{c\pm }\right|$. Moreover, symmetry-breaking phenomena along the *y*-direction exhibit analogous mechanisms to those observed in the *x*-direction, and are consequently omitted from the figure to avoid redundancy.

Through exact analysis in Equations (B3) and (B4) and numerical fitting in Figure 2, we summarize the complete behaviors of the quantum criticality in Table 1. These results are consistent with the phenomena observed in the coupled-top model, as well as in the light–matter interaction systems such as the Rabi and Dicke models, which all exhibit the $Z_{2}$ symmetry [41,47,48].

## 5. Excitation Spectra for U(1) Symmetry

Special attention should be paid to $|\lambda_{x}|=|\lambda_{y}\left|=|\lambda \right|$, which corresponds to the isotropic coupled-top model with U(1) symmetry. Strong spin–spin interaction can break the U(1) symmetry, with critical points $\lambda^{\mp }=\mp 1$. Furthermore, these critical points represent the intersections of three phases: PP, FP, and AFP, as shown in Figure 1a. Each point in the U(1) symmetry-breaking region exhibits infinite degeneracy, associated with the Goldstone mode.

Using the transformations as in the previous section, we can derive a similar form of the quadratic term ${\hat{\tilde{H}}}_{2}$ as in Equations (24) and (27). In the U(1) symmetry-breaking regions, the parameters are $\omega_{1}=\omega_{2}=\left|\lambda \right|$, $\tau_{1,2}=\tau_{2,1}=-\frac{1}{\left|\lambda \right|}$, and $g_{1,2}=g_{2,1}=-k\lambda$, whereas in the symmetry-preserving regions, the parameters become $\omega_{1}=\omega_{2}=1$, $\tau_{1,2}=\tau_{2,1}=-\left|\lambda \right|$, and $g_{1,2}=g_{2,1}=-k\lambda$. The discrete parameter k=±1 serves as an indicator, corresponding to $\lambda_{x}=\lambda_{y}=\lambda$ and $\lambda_{x}=-\lambda_{y}=\lambda$, respectively.

Consequently, for $\lambda^{c-}<\lambda <\lambda^{c+}$, the excitation spectra for U(1) symmetry are given by

(29) $$\Delta_{1}=1+|\lambda |,\;\Delta_{2}=1-\left|\lambda \right|.$$

While in symmetry-breaking regions for $\lambda >\lambda^{c+}$ and $\lambda <\lambda^{c-}$,

(30) $$\Delta_{1}={\left(2+2\lambda^{2}\right)}^{1/2},\;\Delta_{2}=0.$$

**Figure 3:**
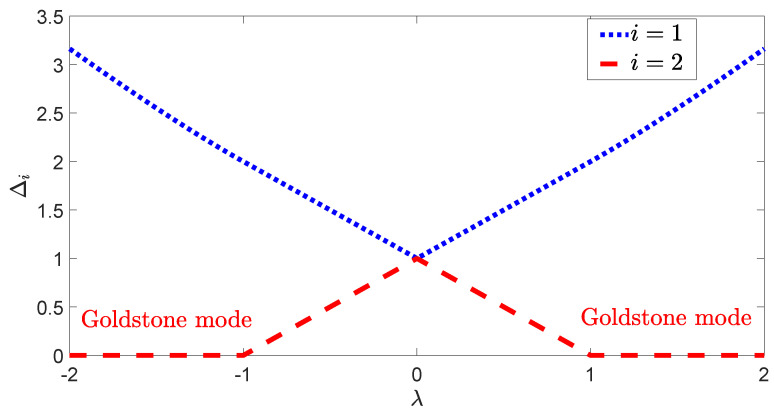
The two branches of excitation energies $\Delta_{i}$ as functions of λ where $\lambda_{x}=\lambda_{y}=\lambda$. The energy gap, which is the minimum of two branches, vanishes beyond the critical points, associated with the Goldstone mode.

As shown in Figure 3, two branches of excitation energies, represented by blue and red dashed lines, intersect at λ=0. In contrast to the dispersions shown in Figure 2a, the energy gap of the lowest branch vanishes beyond the critical points $\lambda^{c\mp }=\mp 1$, consistent with the infinite degeneracy of the Goldstone mode [48,50,68]. Moreover, near the critical points where $\lambda^{c-}\to \lambda$ and $\lambda \to \lambda^{c+}$, the energy gap follows a significantly different exponential relation,

(31) $$\Delta_{min}\propto {\left|\lambda -\lambda^{c\pm }\right|}^{1},$$

where the exponent value is 1. This unusual exponent value has also been found in other U(1)-symmetry-breaking systems, such as the Jaynes–Cummings model [50].

The vanishing energy gap and the corresponding distinct exponent highlight the uniqueness of the continuous symmetry in contrast to the discrete symmetry. The coupled-top model provides an ideal platform for investigations of critical behaviors associated with distinct symmetries.

## 6. Conclusions

In this paper, we investigate the quantum phase transition in the coupled-top model. It describes the interactions between two spin ensembles along both *x*- and *y*-directions, which can be regarded as a generalization of the XY model in a transverse field. Notably, by tuning the coupling constants, the coupled-top model bridges the gap between discrete $Z_{2}$ symmetry and continuous U(1) symmetry, which provides an important platform to study distinct critical behaviors associated with different symmetry breaking.

Using the mean-field approach, we derive the novel phase diagram in the thermodynamic limit J→∞, which consists of five phases: the disordered paramagnetic phase (PP), the ordered ferromagnetic phase with symmetry breaking along *x*- or *y*-direction (*x*-FP or *y*-FP), the ordered antiferromagnetic phase with symmetry breaking along *x*- or *y*-direction (*x*-AFP or *y*-AFP). When the coupling strengths exceed the critical points $\lambda^{c\mp }=\mp 1$ for $|\lambda_{x}\left|\neq \right|\lambda_{y}|$, the discrete $Z_{2}$ symmetry breaking occurs in *d*-direction (d=x,y), depending on the larger one of $|\lambda_{x}|$ and $|\lambda_{y}|$. It results in a double degeneracy with spontaneous magnetization in *x*- or *y*-directions. On the other hand, when $|\lambda_{x}\left|=\right|\lambda_{y}|$, the continuous U(1) symmetry is spontaneously broken beyond the critical points, leading to the emergence of infinite degeneracy associated with the Goldstone mode.

To investigate quantum criticality and fluctuations beyond the mean-field ansatz, we retain and solve the quadratic term in the transformed Hamiltonian using a sequence of transformations. Regarding the $Z_{2}$ symmetry breaking, we present evidence of the second-order quantum phase transitions, specifically from PP to FP and AFP. At the critical points, the excitation energy vanishes, and both quantum fluctuations and entanglement entropy diverge. Furthermore, the analysis of the critical behaviors near the phase transition points confirms the breaking of $Z_{2}$ symmetry. In contrast, when the system undergoes U(1) symmetry breaking, the energy gap vanishes beyond the critical points, yielding a novel exponential relation.

Our approach can be easily extended to systems with multiple large spins, where both anisotropy and geometric frustration are of substantial significance. The competition between them is likely to give rise to intricate quantum phase transitions and critical phenomena, rendering it worthy of further exploration.

## Figures and Tables

**Table 1:** Critical behavior near the phase transition point due to $Z_{2}$ symmetry breaking: the excitation energy $\Delta_{min}$, the quantum fluctuation ${\left(\Delta x_{i}\right)}^{2}$, and the entanglement entropy $S_{i}$ along *d*-direction for d=x,y.

	Ferromagnetic Phase ($\lambda <\lambda_{d}^{c-}$)	Paramagnetic Phase ($\lambda_{d}^{c-}<\lambda <\lambda_{d}^{c+}$)	Antiferromagnetic Phase ($\lambda >\lambda_{d}^{c+}$)
$\Delta_{min}$	${\left(\lambda_{d}^{c-}-\lambda_{d}\right)}^{1/2}$	$|\lambda_{d}-\lambda_{d}^{c\pm }{|}^{1/2}$	${\left(\lambda_{d}-\lambda_{d}^{c+}\right)}^{1/2}$
${\left(\Delta x_{i}\right)}^{2}$	${\left(\lambda_{d}^{c-}-\lambda_{d}\right)}^{-1/2}$	$|\lambda_{d}-\lambda_{d}^{c\pm }{|}^{-1/2}$	${\left(\lambda_{d}-\lambda_{d}^{c+}\right)}^{-1/2}$
$S_{i}$	$-1/4\ln(\lambda_{d}^{c-}-\lambda_{d})$	$-1/4\ln|\lambda_{d}-\lambda_{d}^{c\pm }|$	$-1/4\ln(\lambda_{d}-\lambda_{d}^{c+})$

## Data Availability

The data that support the findings of this study are available from the corresponding author upon reasonable request.

## Abbreviations

The following abbreviations are used in this manuscript:

CTM	coupled-top model
TFIM	transverse-field Ising mode
PP	paramagnetic phase
FP	ferromagnetic phase
AFP	antiferromagnetic phase

## Appendix A. Expansion of the Hamiltonian with HP Transformation

The second term in Equation (22) can be written as

(A1) $$\begin{array}{cc} {\hat{\tilde{H}}}_{1}= & \;\frac{\sqrt{2}}{2}\left({\hat{a}}_{1}^{†}+{\hat{a}}_{1}\right)\left(\sin\theta_{1}+\lambda_{x}\cos\phi_{1}\cos\theta_{1}\sin\theta_{2}\cos\phi_{2}\right)\\ \; & +\lambda_{y}\sin\phi_{1}\cos\theta_{1}\sin\theta_{2}\sin\phi_{2}\\ \; & +\frac{\sqrt{2}}{2}\left({\hat{a}}_{2}^{†}+{\hat{a}}_{2}\right)\left(\sin\theta_{2}+\lambda_{x}\cos\phi_{1}\sin\theta_{1}\cos\theta_{2}\cos\phi_{2}\right)\\ \; & +\lambda_{y}\sin\phi_{1}\sin\theta_{1}\cos\theta_{2}\sin\phi_{2}\\ \; & -\frac{\sqrt{2}}{2i}\left({\hat{a}}_{1}^{†}-{\hat{a}}_{1}\right)\left(-\lambda_{x}\sin\phi_{1}\sin\theta_{2}\cos\phi_{2}+\lambda_{y}\cos\phi_{1}\sin\theta_{2}\sin\phi_{2}\right)\\ \; & -\frac{\sqrt{2}}{2i}\left({\hat{a}}_{2}^{†}-{\hat{a}}_{2}\right)\left(-\lambda_{x}\cos\phi_{1}\sin\theta_{1}\sin\phi_{2}+\lambda_{y}\sin\phi_{1}\sin\theta_{1}\cos\phi_{2}\right). \end{array}$$

According to Equations (8a)–(8d), we can easily prove that ${\hat{\tilde{H}}}_{1}=0$.

The third term in Equation (22) is specifically expressed as

(A2) $$\begin{array}{cc} {\hat{\tilde{H}}}_{2}= & \;\cos\theta_{1}{\hat{a}}_{1}^{†}{\hat{a}}_{1}+\cos\theta_{2}{\hat{a}}_{2}^{†}{\hat{a}}_{2}\\ \; & +\lambda_{x}[\frac{1}{2}\cos\phi_{1}\cos\phi_{2}\cos\theta_{1}\cos\theta_{2}\left({\hat{a}}_{1}^{†}+{\hat{a}}_{1}\right)\left({\hat{a}}_{2}^{†}+{\hat{a}}_{2}\right)\\ \; & +\frac{1}{2i}\cos\phi_{1}\sin\phi_{2}\cos\theta_{1}\left({\hat{a}}_{1}^{†}+{\hat{a}}_{1}\right)\left({\hat{a}}_{2}^{†}-{\hat{a}}_{2}\right)\\ \; & +\frac{1}{2i}\sin\phi_{1}\cos\theta_{2}\cos\phi_{2}\left({\hat{a}}_{1}^{†}-{\hat{a}}_{1}\right)\left({\hat{a}}_{2}^{†}+{\hat{a}}_{2}\right)\\ \; & -\frac{1}{2}\sin\phi_{1}\sin\phi_{2}\left({\hat{a}}_{1}^{†}-{\hat{a}}_{1}\right)\left({\hat{a}}_{2}^{†}-{\hat{a}}_{2}\right)\\ \; & -\sin\theta_{1}\sin\theta_{2}\cos\phi_{1}\cos\phi_{2}\left({\hat{a}}_{1}^{†}{\hat{a}}_{1}+{\hat{a}}_{2}^{†}{\hat{a}}_{2}\right)]\\ \; & +\lambda_{y}[(\frac{1}{2}\sin\phi_{1}\sin\phi_{2}\cos\theta_{1}\cos\theta_{2}\left({\hat{a}}_{1}^{†}+{\hat{a}}_{1}\right)\left({\hat{a}}_{2}^{†}+{\hat{a}}_{2}\right)\\ \; & -\frac{1}{2i}\sin\phi_{1}\cos\phi_{2}\cos\theta_{1}\left({\hat{a}}_{1}^{†}+{\hat{a}}_{1}\right)\left({\hat{a}}_{2}^{†}-{\hat{a}}_{2}\right)\\ \; & -\frac{1}{2i}\cos\phi_{1}\cos\theta_{2}\sin\phi_{2}\left({\hat{a}}_{1}^{†}-{\hat{a}}_{1}\right)\left({\hat{a}}_{2}^{†}+{\hat{a}}_{2}\right)\\ \; & -\frac{1}{2}\cos\phi_{1}\cos\phi_{2}\left({\hat{a}}_{1}^{†}-{\hat{a}}_{1}\right)\left({\hat{a}}_{2}^{†}-{\hat{a}}_{2}\right)\\ \; & -\sin\theta_{1}\sin\theta_{2}\sin\phi_{1}\sin\phi_{2}\left({\hat{a}}_{1}^{†}{\hat{a}}_{1}+{\hat{a}}_{2}^{†}{\hat{a}}_{2}\right)], \end{array}$$

which has a quadratic form and can be solved exactly by the symplectic transformation [64].

## Appendix B. The Excitation Energy Δ*_i_* and Quantum Fluctuations (Δ*x_i_*)^2^, (Δ*p_i_*)^2^ Obtained from Symplectic Transformation *S*

According to Equation (26), we derive the general form of the symplectic transformation *S* as follows:

(B1) $$\begin{array}{c} S=\sqrt{\frac{1}{2}}\left(\begin{array}{cccc} \alpha_{+}^{-1} & \alpha_{-}^{-1} & 0 & 0\\ -\alpha_{+}^{-1} & \alpha_{-}^{-1} & 0 & 0\\ 0 & 0 & \alpha_{+} & \alpha_{-}\\ 0 & 0 & -\alpha_{+} & \alpha_{-} \end{array}\right) \end{array}$$

where

(B2) $$\begin{array}{c} \alpha_{+}={\left(\frac{\omega +|\tau |}{\omega +|g|}\right)}^{\frac{1}{4}},\;\alpha_{-}={\left(\frac{\omega -|\tau |}{\omega -|g|}\right)}^{\frac{1}{4}}. \end{array}$$

In the symmetry-breaking regions along *d*-direction (d=x,y), where $\lambda_{d}<\lambda_{d}^{c-}$ and $\lambda_{d}>\lambda_{d}^{c+}$, under the condition $|\lambda_{d}>\left|\lambda_{d^{\prime }}\right|$ with the the orthogonal direction $\left(d^{\prime }=y,x\right)$, we can analytically compute the the excitation energy $\Delta_{i}$, quantum fluctuations ${\left(\Delta x_{i}\right)}^{2}$ and ${\left(\Delta p_{i}\right)}^{2}$ as

(B3a) $$\begin{array}{cc} \Delta_{1} & ={\left[\left(\right|\lambda_{d}|+\frac{1}{|\lambda_{d}|})(|\lambda_{d}\left|+\right|\lambda_{d^{\prime }}\left|\right)\right]}^{1/2}, \end{array}$$

(B3b) $$\begin{array}{cc} \Delta_{2} & ={\left[\left(\right|\lambda_{d}|-\frac{1}{|\lambda_{d}|})(|\lambda_{d}\left|-\right|\lambda_{d^{\prime }}\left|\right)\right]}^{1/2}, \end{array}$$

(B3c) $$\begin{array}{cc} {\left(\Delta x_{i}\right)}^{2} & =\frac{1}{4}\left[{\left(\frac{\lambda_{d}^{2}-\left|\lambda_{d}\lambda_{d^{\prime }}\right|}{\lambda_{d}^{2}-1}\right)}^{1/2}+{\left(\frac{\lambda_{d}^{2}+\left|\lambda_{d}\lambda_{d^{\prime }}\right|}{\lambda_{d}^{2}+1}\right)}^{1/2}\right], \end{array}$$

(B3d) $$\begin{array}{cc} {\left(\Delta p_{i}\right)}^{2} & =\frac{1}{4}\left[{\left(\frac{\lambda_{d}^{2}-1}{\lambda_{d}^{2}-\left|\lambda_{d}\lambda_{d^{\prime }}\right|}\right)}^{1/2}+{\left(\frac{\lambda_{d}^{2}+1}{\lambda_{d}^{2}+\left|\lambda_{d}\lambda_{d^{\prime }}\right|}\right)}^{1/2}\right]. \end{array}$$

While in the symmetry-preserving paramagnetic phase for $\lambda_{d}^{c-}<\lambda_{d}<\lambda_{d}^{c+}$, they are expressed as

(B4a) $$\begin{array}{cc} \Delta_{1} & ={\left[\left(1+\right|\lambda_{d}\left|)(1+\right|\lambda_{d^{\prime }}\left|\right)\right]}^{1/2}, \end{array}$$

(B4b) $$\begin{array}{cc} \Delta_{2} & ={\left[\left(1-\right|\lambda_{d}\left|)(1-\right|\lambda_{d^{\prime }}\left|\right)\right]}^{1/2}, \end{array}$$

(B4c) $$\begin{array}{cc} {\left(\Delta x_{i}\right)}^{2} & =\frac{1}{4}\left[{\left(\frac{1-|\lambda_{d^{\prime }}|}{1-|\lambda_{d}|}\right)}^{1/2}+{\left(\frac{1+|\lambda_{d^{\prime }}|}{1+|\lambda_{d}|}\right)}^{1/2}\right], \end{array}$$

(B4d) $$\begin{array}{cc} {\left(\Delta p_{i}\right)}^{2} & =\frac{1}{4}\left[{\left(\frac{1-|\lambda_{d}|}{1-|\lambda_{d^{\prime }}|}\right)}^{1/2}+{\left(\frac{1+|\lambda_{d}|}{1+|\lambda_{d^{\prime }}|}\right)}^{1/2}\right]. \end{array}$$
